# Gallic Acid-L-Leucine Conjugate Protects Mice against LPS-Induced Inflammation and Sepsis via Correcting Proinflammatory Lipid Mediator Profiles and Oxidative Stress

**DOI:** 10.1155/2018/1081287

**Published:** 2018-03-25

**Authors:** Yuanyuan Cheng, Xuechen Li, Hung-Fat Tse, Jianhui Rong

**Affiliations:** ^1^School of Chinese Medicine, The University of Hong Kong, 10 Sassoon Road, Pokfulam, Hong Kong; ^2^Department of Chemistry, The University of Hong Kong, Pokfulam Road, Pokfulam, Hong Kong; ^3^Department of Medicine, The University of Hong Kong, 21 Sassoon Road, Pokfulam, Hong Kong

## Abstract

The pathology of endotoxin LPS-induced sepsis is hallmarked by aberrant production of proinflammatory lipid mediators and nitric oxide (NO). The aim of the present study was to determine whether the new product gallic acid-L-leucine (GAL) conjugate could ameliorate the LPS-induced dysregulation of arachidonic acid metabolism and NO production. We first investigated the effects of GAL conjugate on the expression of proinflammatory enzymes and the production of proinflammatory NO and lipid mediators in mouse macrophage cell line RAW264.7, primary peritoneal macrophages, and mouse model. Western blot analyses revealed that GAL attenuated LPS-induced expression of iNOS, COX-2, and 5-LOX in a concentration-dependent manner. Consistently, probing NO-mediated fluorescence revealed that GAL antagonized the stimulatory effect of LPS on iNOS activity. By profiling of lipid mediators with ESI-MS-based lipidomics, we found that GAL suppressed LPS-induced overproduction of prostaglandin E2, prostaglandin F2, leukotriene B4, and thromboxane B2. We further discovered that GAL might exhibit anti-inflammatory activities by the following mechanisms: (1) suppressing LPS-induced activation of MAP kinases (i.e., ERK1/2, JNK, and p38); (2) reducing the production of reactive oxygen species (ROS); and (3) preventing LPS-induced nuclear translocation of transcription factors NF-*κ*B and AP-1. Consequently, GAL significantly decreased the levels of COX-2 and iNOS expression and the plasma levels of proinflammatory lipid mediators in LPS-treated mice. GAL pretreatment enhanced the survival of mice against LPS-induced endotoxic shock. Taken together, our results suggest that GAL may be a potential anti-inflammatory drug for the treatment of endotoxemia and sepsis.

## 1. Introduction

Microbial infection and tissue injury often induce various inflammatory responses involving the infiltration of polymorphonuclear leukocytes/macrophages and the production of a battery of proinflammatory mediators [1, 2]. Excessive production of cytotoxic mediators directly damages the surrounding tissues, leading to various pathological consequences. Endotoxic lipopolysaccharide (LPS) is widely used to activate macrophages in cell and animal models for evaluating potential anti-inflammatory drugs [3]. LPS is well-known to activate the transcription factors such as nuclear factor *κ*B (NF-*κ*B) and activator protein-1 (AP-1) via inducing the phosphorylation of the inhibitor-*κ*B (I-*κ*B) and/or mitogen-activated protein kinases (MAPKs) in macrophages [4, 5]. Upon inflammatory activation, transcription factors NF-*κ*B, CREB, C/EBP, and AP-1 induce the expression of cyclooxygenase-2 (COX-2), 5-lipoxygenase (5-LOX), and inducible NO synthase (iNOS) gene in macrophages [6, 7]. These proinflammatory enzymatic products including NO, prostaglandins, leukotrienes, and thromboxanes contribute the regulation of heat, pain, and redness [8].

**Figure 1:**
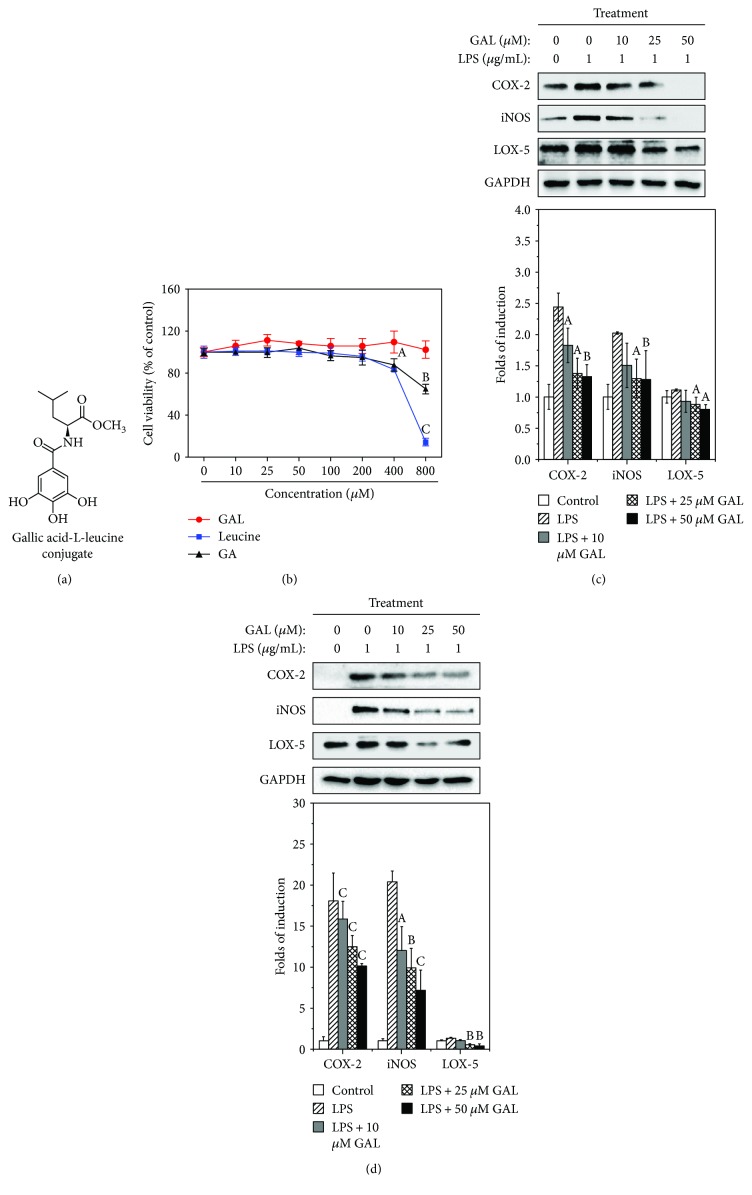
Effects of gallic acid-L-leucine (GAL) conjugate on the cell growth and expression of COX-2, 5-LOX, and iNOS. (a) Chemical structure of gallic acid derivative GAL. (b) Effect of GAL on the cell viability. After 72 h treatment, the cell viability of RAW264.7 cells was determined by colorimetric MTT assay. The values represent the mean ± SD of three experimental replicates. (c) Effects of GAL on LPS-induced COX-2, 5-LOX, and iNOS in RAW264.7 cells. Following 24 h treatment with GAL and LPS, the expression of COX-2, 5-LOX, and iNOS was analyzed by Western blotting and quantified by a densitometric method. The results were expressed as a percentage of the untreated control (*n* = 3). ^a^ *p* < 0.05; ^b^ *p* < 0.01 (sample versus LPS alone). (d) Effect of GAL on LPS-induced COX-2, 5-LOX, and iNOS in primary macrophages. The expression of COX-2, 5-LOX, and iNOS was analyzed in the same fashion as described in (b). The results were expressed as a percentage of the untreated control (*n* = 3). ^b^ *p* < 0.01; ^c^ *p* < 0.001 (sample versus LPS alone).

Herbal medicines are known to modulate proinflammatory enzymes (e.g., COX, LOX, and phospholipase A2 (PLA2)) in arachidonic acid metabolism [9–11]. As a common botanical compound, gallic acid not only scavenges free radicals but also regulates different intracellular signaling pathways [12]. On the other hand, amino acid-L-leucine could regulate cell survival, proliferation, and apoptosis via targeting the mechanistic target of rapamycin (mTOR) signaling pathways [13, 14]. We recently attempted to integrate the chemical features of gallic acid and methyl-L-leucine to yield a novel gallic acid-L-leucine (GAL) conjugate (Figure 1(a)). We indeed discovered that GAL could promote the phagocytosis in macrophages possibly via inducing leukotriene B4 12-hydroxydehydrogenase (LTB4DH) [15]. These results suggest that GAL conjugate may serve as a lead compound for the development of new anti-inflammatory drugs.

In the present study, we focused on the effects of GAL on the production of proinflammatory NO and lipid mediators in macrophages and mice against LPS challenge. We further explored the molecular mechanism underlying the anti-inflammatory activity of GAL through monitoring the activation of MAPKs and NF-*κ*B/AP-1 pathways.

## 2. Materials and Methods

### 2.1. Materials

Antibodies against ERK1/2, phospho-ERK1/2, Akt, phospho-Akt, c-Jun N-terminal kinases (JNK), phospho-JNK, p38, phospho-p38, COX-2, 5-LOX, glyceraldehydes-3-phosphate dehydrogenase (GAPDH), *β*-actin, lamin b, and Alexa Fluor 594 conjugated goat anti-rabbit IgG antibodies were obtained from Cell Signaling Technology (Boston, MA, USA). Anti-NF-*κ*B and anti-mouse horseradish peroxidase- (HRP-) conjugated IgG antibodies were purchased from Santa Cruz Biotechnology (Santa Cruz, CA, USA). Dihydroethidium (DHE) was purchased from molecular probes (Eugene, OR, USA). Dexamethasone was obtained from Santa Cruz Biotechnology (Santa Cruz, CA, USA). Anti-rabbit HRP-conjugated IgG secondary antibody and other chemicals were purchased from Sigma-Aldrich (St. Louis, MO, USA), unless otherwise indicated. Gallic acid-l-leucine (GAL) conjugate was synthesized from gallic acid and methyl-l-leucine hydrochloride by following the previous method as described [15]. The structure of GAL conjugate was confirmed by NMR technique whereas the purity of the product was determined by HPLC analysis, suggesting the purity of up to 98%.

### 2.2. Cell Culture and Drug Treatment

RAW264.7 cells were obtained from Shanghai Institute for Biological Sciences, Chinese Academy of Science (Shanghai, China) and cultured in Dulbecco modified Eagle medium (DMEM) supplemented with 2 mM glutamine, antibiotics (100 U/ml of penicillin A and 100 U/ml of streptomycin), and 10% heat-inactivated fetal bovine serum (Gibco/BRL, Gaithersburg, MD, USA) and maintained at 37°C in a humidified incubator containing 5% CO_2_. For drug treatment, RAW264.7 cells were seeded at the density of 1.0 × 10^5^ in 6-well plate overnight and pretreated with the conjugate GAL for 2 h and stimulated with 1 *μ*g/ml of LPS for another 24 h.

### 2.3. Animal Care

The protocols for animal experiments were approved by the Committee on the Use of Live Animals in Teaching and Research, The University of Hong Kong. Male C57BL/6 mice (6–8 weeks old) were obtained from the Laboratory Animal Unit, The University of Hong Kong. C57BL/6 mice were kept in a conditional room with controlled temperature (20–25°C), relative humidity (60–70%), and day/night cycle (12 : 12 light/dark).

### 2.4. Isolation of Mouse Peritoneal Macrophages

The primary peritoneal macrophages were isolated from male C57BL/6 mice (6–8 weeks old) as previously described [16]. After i.p injection of 3 ml 3% thioglycollate broth (Fluka, MO, USA) for 3 days, peritoneal lavage was carried out by injecting 5 ml of sterile 3% FBS in phosphate-buffer saline (PBS). The cells were collected and pelleted by centrifugation at 1200 rpm for 8 min. The cell pellet was suspended in 10% FBS in DMEM and incubated in cell culture dishes for 2 h. After the removal of the nonadherent cells, the adherent cells were recovered as primary macrophages.

### 2.5. Cell Viability Assay

The cytotoxicity of GAL conjugate was evaluated by a colorimetric assay using 3-(4, 5-dimethyl-2-thiazolyl)-2, 5-diphenyl-2H-tetrazolium bromide (MTT). Briefly, following 72 h treatment with GAL conjugate at the concentrations of 0, 10, 25, 50, 100, 200, 400, and 800 *μ*M, MTT was added to the cell culture of mouse macrophage RAW264.7 cells in a 96-well plate (final concentration of 0.5 mg/ml). After the incubation at 37°C under 5% CO_2_ for 4 h, the supernatant was removed and the resulted formazan crystals in viable cells were solubilized with 150 *μ*l of dimethyl sulfoxide (DMSO) and measured on a Bio-Red microplate reader (Hercules, CA, USA) at 570 nm.

### 2.6. Western Blotting Analysis of Protein Levels

The cellular proteins were extracted by using ice-cold RIPA buffer (20 mM Tris-HCl, 150 mM NaCl, 1 mM Na_2_EDTA, 1 mM EGTA, 1% NP-40, 1% sodium deoxycholate, 2.5 mM sodium pyrophosphate, 1 mM beta-glycerophosphate,1 mM Na3VO4, 1 *μ*g/ml leupeptin, and pH 7.5). To prepare proteins from mouse tissues, male C57BL/6 (6–8 weeks) mice were randomly divided into three groups, control, receiving vehicle only; LPS, receiving 4 mg/kg; and LPS + GAL (i.e., 2.5, 5.0, and 10.0 mg/kg), where mice were pretreated with GAL via i.p injection for 3 days and subsequently challenged with LPS via i.p injection at the time of 12 h after the injection of GAL on day 3. After LPS stimulation for another 12 h, the spleens were collected from the animals in each experimental group. The proteins were extracted from primary peritoneal macrophages and spleen tissues by using ice-cold RIPA buffer (20 mM Tris-HCl, 150 mM NaCl, 1 mM Na_2_EDTA, 1 mM EGTA, 1% NP-40, 1% sodium deoxycholate, 2.5 mM sodium pyrophosphate, 1 mM beta-glycerophosphate,1 mM Na_3_VO_4_, and 1 *μ*g/ml leupeptin, pH 7.5). Thirty micrograms of proteins was resolved on 10% SDS-PAGE and subsequently transferred onto PVDF transfer membrane (EMD Millipore, Billerica, MA, USA). Following the incubation in 5% nonfatted milk powder in TBST (50 mM Tris-Cl, 150 mM NaCl, and 0.1% Tween-20, pH 7.6) at 4°C for 4 h, the blots were probed with the antibodies specific for COX-2, iNOS, and GAPDH (1 : 1000 dilution) at 4°C overnight. The bound antibodies were detected by horseradish peroxidase- (HRP-) conjugated secondary antibody (1 : 1000 dilution) at 4°C for 3 h and then visualized by enhanced chemiluminescence (ECL) reaction reagents (GE Healthcare, Uppsala, Sweden). The protein levels of individual proteins on Western blots were determined by a densitometric method.

### 2.7. Reverse Transcription Polymerase Chain Reaction (RT-PCR) Detection

The cells were treated with GAL, NF-*κ*B inhibitor, AP-1 inhibitors, and LPS as indicated in individual experiments. The total RNAs were isolated with Trizol reagent (Invitrogen, CA, USA) and converted into corresponding cDNAs using SuperScript III reverse transcriptase and random hexamer primers (Thermo, Waltham, MA, USA). The mRNA expression was detected with specific primers as follows: COX-2 mRNA (NM_011198), sense: 5′-TGATCGAAGACTACGTGCAAC-3′, antisense: 5′-TCATCTCTCTGCTCTGGTCAA-3′; 5-LOX mRNA (NM_009662), sense: 5′-CCCCCGAAGCTCCCAGTGACC-3′, antisense: 5′-TCCCGGGCCTTAGTGTTGATA-3′; iNOS mRNA (NM_010927), sense: 5′-AAAGTGACCTGAAAGAGGAAAAGGA-3′, antisense: 5′-TTGGTGACTCTTAGGGTCATCTTGTA-3′; *β*-actin mRNA (NM_007393), sense: 5′-ATGGATGACGATATCGCTGC-3′, antisense: 5′-TTCTGACCCATTCCCACCATC-3′. PCR amplifications were performed according to the following program: denaturation at 94°C for 3 min, 25 cycles, (94°C for 30 sec, 57°C for 30 sec, and 72°C for 1 min) and extension at 72°C for 10 min. PCR products were analyzed by gel electrophoresis in 1.5% agarose containing GelRed nucleic acid (Biotium, Hayward, CA, USA) and visualized under UV light.

### 2.8. Lipidomics Profiling of Arachidonic Acid Metabolites

For lipid preparation, the cell culture medium and cells were treated with methanol at the final concentration of 15% (*v*/*v*), supplemented with 40 ng of freshly prepared internal standards (LTB4-d_4_). After the proteins were precipitated and removed by centrifugation at 4°C, 5000 rpm, for 10 min, the clear supernatants were transferred into clean 15 ml Falcon tubes and acidified to pH 3 by gradually adding 1 M hydrogen chloride (HCl). The lipid mediators were purified on SPE C18 columns (Cayman, Michigan, USA) according to the manufacturer's instruction. The lipids were eluted with 12 ml of methyl formate. After being dried under nitrogen and in the dark (to prevent photo degradation), the lipid residues were dissolved in 60 *μ*l of ethanol/water (70/30, *v*/*v*).

For lipid preparation from plasma, the plasma samples were collected from the animals treated with vehicle, LPS, or LPS + GAL. The lipid mediators were isolated similarly as described for lipid preparation from the cells.

Lipids were separated on a Synergi hydro-RP C18 reversed phase column (150 mm × 4.6 mm, 4 *μ*m) from Phenomenex (Torrance, CA, USA). For HPLC separation, an Agilent HLPC 1100 system (Santa Clara, CA, USA) is equipped with a binary pump, a microvacuum degasser, a column compartment with thermostat control, and a temperature-controlled HTC PAL autosampler (CTC Analytics, Zwingen, Switzerland). The composition of mobile phase was (A) water with 0.05% (*v*/*v*) formic acid and (B) acetonitrile with 0.05% (*v*/*v*) formic acid. Gradient was set as follows: 0–2.5 min, 40–50% B; 2.5–5.5 min, 50–80% B; 5.5–11.5 min, 80% B; and 11.5–12 min, 80–40% B, 12–15 min, 40% B. Flow rate was constant at 0.7 ml/min. The column temperature was maintained at 30°C. Sample volume for injection was 20 *μ*l. The elution was monitored by an ABI/Sciex triple quadrupole mass spectrometer 3200 QTRAPLC/MS/MS system (Applied Biosystems/MDS Sciex, Concord, ON, Canada) equipped with an ESI-Turbo V source operating in negative ionization mode that was used for analysis. The system was controlled by Analyst v1.4.2 data system (Applied Biosystems/MDS Sciex, Concord, ON, Canada). MS analysis was conducted in electrospray negative mode. The mass spectrometer was operated with ion spray voltage of −4.5 kV with 325°C drying gas, 40 psi nebulizer gas, and 60 psi turbo gas. Multiple-reaction monitoring (MRM) detection was used for the quantitation of all analytes. The collision-induced-dissociation parameters were optimized, and MRM pairs were selected by continuous infusion of internal standards at the concentration of 100 nM and at the flow rate of 10 *μ*l/min with Harvard infusion pump (Harvard Apparatus GmbH, March, Germany). The MS profile of lipid detection was provided in Supplementary Figure 3 and Table 1.

### 2.9. Measurement of NO and Reactive Oxygen Species (ROS)

Primary peritoneal macrophages or RAW264.7 cells were seeded at the density of 1 × 10^6^ cells/well in confocal dish. For the detection of intracellular NO production, the cells were pretreated with GAL (0 or 50 *μ*M) for 2 h and subsequently stimulated with 1 *μ*g/ml LPS for 24 h. Following LPS stimulation, the cells were incubated with 5 *μ*M DAF-FM diacetate (4-amino-5-methylamino-2′,7′-difluorofluorescein diacetate) (Life Technologies, NY, USA) at 37°C for 30 min. After the removal of excessive probe, the fluorescence intensity was determined on a laser scanning microscope (Carl-Zeiss, Jena, Germany). The results were expressed as a percentage relative to the intracellular NO production in the cells stimulated with LPS alone. As for the measurement of NO level in cell culture medium, the culture medium was collected and detected by Griess reagent kit (Thermo Fisher Scientific, MA USA).

On the other hand, the intracellular ROS was assessed using 2′,7′-dichlorofluorescein-diacetate (DCFH2-DA) (Invitrogen, Grand Island, NY, USA), whereas the superoxide ions were visualized with DHE (Molecular Probes, Eugene, OR). Briefly, RAW264.7 cells were seeded at 1 × 10^6^ cells/well in confocal dishes for 24 h. Following the pretreatment with GAL (0, 10, 25, and 50 *μ*M) for 2 h, the cells were stimulated with LPS for 24 h. At the end of the stimulation, the cells were incubated with 10 *μ*M DCFH2-DA and 2 *μ*M DHE at 37°C for 30 min. The excessive probes were removed with PBS. The fluorescence intensity was measured on a Zeiss laser scanning microscope (Carl-Zeiss, Jena, Germany). The results were expressed as a percentage relative to the intracellular ROS production in the cells stimulated with LPS alone.

### 2.10. Transwell Assay of Macrophage Chemotaxis

For the isolation of primary peritoneal macrophages, male C57BL/6 mice (6–8 weeks old) were treated by i.p injection of 3 ml 3% thioglycollate broth (Fluka, MO, USA) for 3 consecutive days. Peritoneal lavage was carried out using 5 ml of sterile phosphate-buffer saline (PBS) containing 3% FBS. The cells were harvested by centrifugation at 1200 rpm for 8 min and suspended in DMEM with 10% FBS. After 2 h incubation, the nonadherent cells were removed and the adherent cells were used as primary macrophages [16].

For the preparation of the conditioned media, primary peritoneal macrophages were treated with 1 *μ*g/ml LPS and 50 *μ*M GAL conjugate, alone or in combination. After 24 h of drug treatment, the cell culture media were recovered as the conditioned media.

Macrophage chemotaxis was measured in a 24-well Transwell plate as described [17, 18]. Briefly, the conditioned medium was placed in the lower chamber of the specific Transwell. Freshly isolated primary peritoneal macrophages (1 × 10^6^ cells/ml) were added onto the polycarbonate membrane in the upper chamber. The cells were allowed to migrate through the membrane with a pore size of 5 *μ*m. After 4 h incubation, primary peritoneal macrophages were removed from inside the upper chamber. On the other hand, the cells that migrated through the membrane were fixed by methanol for 20 min and stained with 0.1% crystal violet for 15 min. After the removal of excessive dye, the membranes were carefully placed onto the glass slide and imaged under a microscopy (Carl-Zeiss, Jena, Germany). After imaging, the membranes were washed with 30% glacial acetic acid for the determination of macrophage migration in a Bio-Rad microplate reader (Hercules, CA, USA) at the wavelength of 570 nm.

### 2.11. Preparation of Nuclear and Cytosol Extracts

The cytosolic and nuclear fractions of the cellular proteins were isolated from LPS- and drug-treated cells as described previously [19]. Briefly, the cell pellets were resuspended in ice-cold Buffer A (pH 8.0, 20 mM N-2-hydroxyethylpiperazine-N′-2-ethanesulfonic acid (HEPES), 1 mM ethylenediaminetetraacetic acid (EDTA), 1.5 mM MgCl_2_, 10 mM KCl, 1 mM DTT, 1 mM sodium orthovanadate, 1 mM NaF, 1 mM PMSF, and 1% (*v*/*v*) protein inhibitor cocktail) for 30 min and subsequently lysed by adding the appropriate volume of 10% (*v*/*v*) NP-40 to a final concentration of 0.625% (*v*/*v*) while vortexing. By centrifugation at 10,000 ×g, the cell lysates were separated into the supernatant as cytosolic extract and the nuclear pellet. The nuclear pellets were incubated in ice-cold Buffer B (pH 8.0, 20 mM HEPES, 1 mM EDTA, 1.5 mM MgCl_2_, 10 mM KCl, 1 mM DTT, 1 mM sodium orthovanadate, 1 mM NaF, 1 mM PMSF, 1% (*v*/*v*) protein inhibitor cocktail, and 20% (*v*/*v*) glycerol for 30 min) and subsequently centrifuged at 15,000 ×g for 5 min. The supernatant was collected as nuclear extract.

### 2.12. Immunofluorescence Analysis

The nuclear translocation of NF-*κ*B p65 was analyzed by fluorescence microscopy as previously described [20]. Briefly, the cells were seeded in confocal dish overnight, incubated with GAL at the indicated concentrations for 2 h prior to the stimulation by 1 *μ*g/ml LPS for another 2 h. Treated cells were fixed and incubated with anti-NF-*κ*B p65 primary antibody at 4°C overnight. After several washes with PBS, the bound antibodies were detected by FITC-labeled goat anti-rabbit immunoglobulin G (IgG) (Invitrogen, Grand Island, NY, USA) at room temperature for 2 h. The cell nuclei were stained with 4′,6-diamidino-2-phenylindole (DAPI; Vector Laboratories) at room temperature for 15 min. The cells were examined on a laser scanning microscope (Carl-Zeiss, Jena, Germany).

### 2.13. Examination of In Vivo Protection against LPS-Induced Endotoxic Shock

Male C57BL/6 (6–8 weeks) mice were randomly divided into three groups, control, receiving vehicle only; LPS, sequentially receiving vehicle for 3 days and 25 mg/kg LPS; and LPS + GAL (10 mg/kg), sequentially receiving 10 mg/kg GAL for 3 days and 25 mg/kg LPS. In practical, mice were pretreated with GAL or vehicle via i.p injection for 3 days and subsequently challenged with LPS via i.p injection at the time of 12 h after the injection of GAL or vehicle on day 3. To examine the effect of GAL on the survival of animals in the LPS-induced model of endotoxin shock, animals were closely examined every 12 h over the period of 10 consecutive days as previously described [21]. The number of dead mice was counted. Survival rate was calculated based on the numbers of alive and dead animals. The animals were sacrificed on day 10 after LPS challenge. The spleens were weighted and examined under a microscope. To examine the short-time effects, mice were randomly divided into four groups: control, receiving equal volume of vehicle; LPS alone, receiving LPS (4 mg/kg); LPS + GAL, receiving LPS (4 mg/kg) and GAL (2.5, 5, 10 mg/kg); and positive control, receiving Dexamethasone (5 mg/kg). Mice were treated with GAL and LPS in the same way as described above. The spleens, residential peritoneal macrophages, and blood samples were collected from each treatment group 12 hours after LPS injection.

### 2.14. Statistical Analysis

The results were presented as means ± SD from three independent experiments. Statistical analysis was performed by ANOVA, followed by LSD test with SPSS 13.0 software (SPSS, Chicago, USA). A *p* value < 0.05 was considered as statistically significant.

## 3. Results

### 3.1. GAL Suppressed LPS-Stimulated Expression of COX2, 5-LOX, and iNOS

To compare the cytotoxicity of GAL, gallic acid, and L-leucine, RAW264.7 cells were treated with GAL, gallic acid, or L-leucine at the concentrations ranging from 10 to 800 *μ*M for 72 h treatment. The cell viability was determined by colorimetric MTT assay. As shown in Figure 1(b), GAL did not show much cytotoxicity in macrophages, whereas gallic acid and methyl-L-leucine showed evident toxicity at higher concentrations (e.g., 800 *μ*M).

To examine the anti-inflammatory potential of GAL, we first employed Western blotting technique to investigate the effects of GAL on the expression of COX-2, 5-LOX, and iNOS in LPS-stimulated macrophages. In practical, RAW264.7 cells and primary peritoneal macrophages were pretreated with GAL at various concentrations for 2 h. The cells were challenged with 1 *μ*g/ml LPS over a period of 24 h. As shown in Figure 1(c), GAL inhibited LPS-induced expression of COX-2, iNOS in a concentration-dependent manner, while showing lesser effect on 5-LOX expression in RAW264.7 cells. Such activity of GAL was confirmed in primary peritoneal macrophages (Figure 1(d)). Moreover, we also compared the effects of gallic acid, methyl-leucine only, and GAL at the same concentration of 50 *μ*M on the expression COX-2, 5-LOX, and iNOS in LPS-stimulated macrophages. GAL showed stronger inhibitory effects than GA and methyl-leucine alone (see Supplementary Fig.  1).

### 3.2. GAL Reduced the Generation of Proinflammatory NO and Lipid Mediators

**Figure 2:**
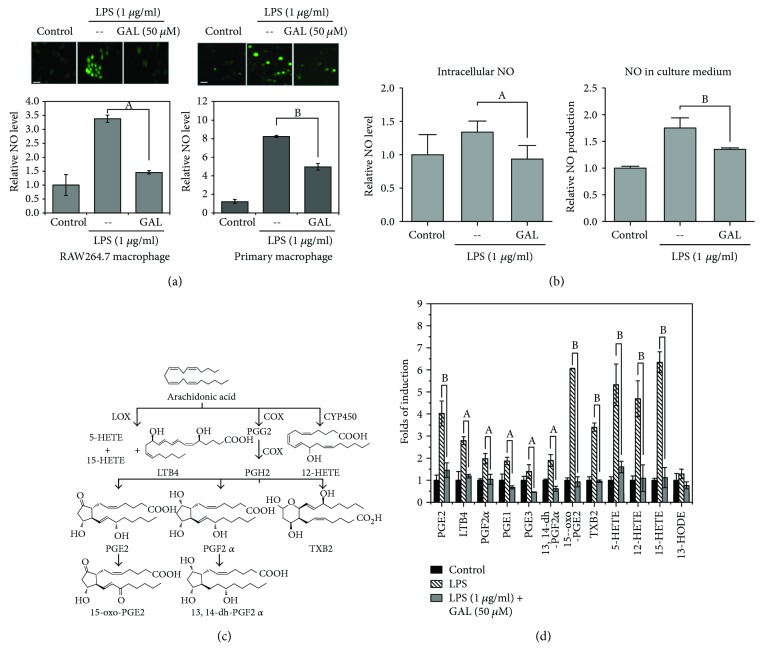
Effects of GAL on the production of proinflammatory mediators (i.e., NO and lipid mediators). (a) Effect of GAL on LPS-induced NO production in RAW264.7 cell and primary macrophages. Following 24 h treatment with LPS and GAL, the intracellular NO was detected by fluorescent probe DAF-FM diacetate and subsequently imaged on a Zeiss fluorescence microscopy (Zeiss, Jena, Germany). Representative images were shown and quantified by Image J software (http://imagej.nih.gov/ij/). The results represent the mean ± SD of three independent experiments. ^a^ *p* < 0.05; ^b^ *p* < 0.01 (sample versus LPS alone); scale bar, 20 *μ*m. (b) Effect of GAL on LPS-induced NO production in RAW264.7 cell and culture medium. Following 24 h treatment with LPS and GAL, the intracellular NO was detected by fluorescent probe DAF-FM diacetate on Fusion™*α*-FP microplate reader (Packard BioScience Company, USA). The NO levels in cell culture medium were assayed by using Griess reagent kit (Thermo Fisher Scientific, MA USA). (c) Scheme illustrating the metabolic pathways for the generation of lipid mediators. (d) LC-MS/MS determination of proinflammatory lipid mediators. After the treatments with GAL and LPS, the lipids were isolated from RAW264.7 cells and culture medium and subsequently quantified by a MRM method on a LC-MS/MS system.

To examine the effects of GAL on the enzymatic products of iNOS, COX-2, and 5-LOX, we first determined the effects of GAL on LPS-induced NO production in RAW264.7 cells and primary peritoneal macrophages by a fluorescence probe DAF-FM diacetate. In practical, RAW264.7 cells and primary peritoneal macrophages were pretreated with GAL (50 *μ*M) for 2 h and subsequently stimulated with LPS for another 24 h. Intracellular NO contents were visualized by direct incubation with DAF-FM diacetate. As shown in Figure 2(a), LPS increased NO production in RAW264.7 cells by ~2.5-fold while in primary peritoneal macrophages by ~8.5-fold relative to that of untreated controls. GAL pretreatment significantly diminished LPS-induced production of intracellular NO in both cell types. As shown in Figure 2(b), we also found that GAL could suppress NO production in the culture medium. On the other hand, we also determined the production of the well-documented lipid mediators from arachidonic acid metabolism as illustrated in Figure 2(c). Following drug treatment and LPS stimulation, several major proinflammatory lipid mediators were quantified by ESI-MS-based lipidomics techniques. As shown in Figure 2(d), LPS differentially stimulated the production of PGE1, PGE2, PGE3, PGF2*α*, TXB2, and LTB4 in macrophages. Importantly, GAL markedly suppressed the stimulatory effects of LPS on the production of the commonly recognized proinflammatory lipid mediators.

### 3.3. GAL Reduced the Chemotactic Potential of Macrophages

**Figure 3:**
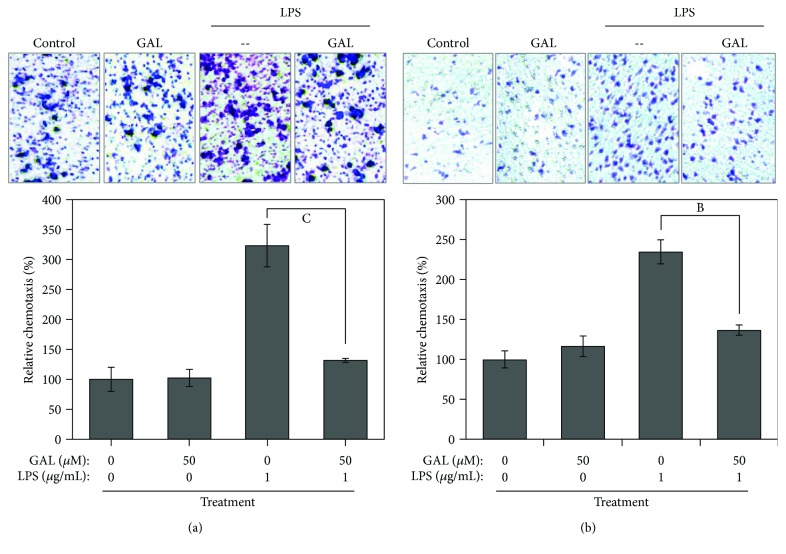
Effects of GAL on the chemotactic potential of macrophages. (a) Effect of GAL on the chemotactic potential of RAW264.7 cells. After 24 h treatment with GAL and LPS, the cell culture medium was recovered and assayed for the chemotactic potential in Transwell assay. The cells that migrated through Transwell membrane were sequentially fixed, stained, and imaged under a microscope. Representative images were shown. Macrophage migration was quantified by measuring the absorbance of the cell lysates. The results represent the means ± SD of three separate experiments. ^a^ *p* < 0.05 (GAL-LPS versus LPS alone). (b) Effect of GAL on the chemotactic potential of primary peritoneal macrophages. The chemotactic potential of primary peritoneal macrophages was analyzed as described.

To examine the effect of GAL on the chemotactic activity of macrophages, RAW264.7 cells and primary peritoneal macrophages were pretreated with 50 *μ*M GAL for 2 h and subsequently stimulated with LPS for another 24 h. The cell culture media were recovered as the conditioned medium for assaying the capacity to attract new unstimulated macrophages. As shown in Figure 3(a), LPS increased the chemotactic potential of the conditioned medium to induce the migration of RAW264.7 cells through Transwell membrane by approximately 2-fold relative to the unstimulated medium. GAL pretreatment antagonized the stimulatory effect of LPS on macrophage migration by approximately 50%. The chemotactic potential of primary macrophages was also assayed and shown in Figure 3(b). GAL showed similar activity against LPS-induced increase in the chemotactic potential of primary peritoneal macrophages.

### 3.4. GAL Protected Mice against Endotoxin Shock and Inhibited the Production of Proinflammatory Factors in Mice

**Figure 4:**
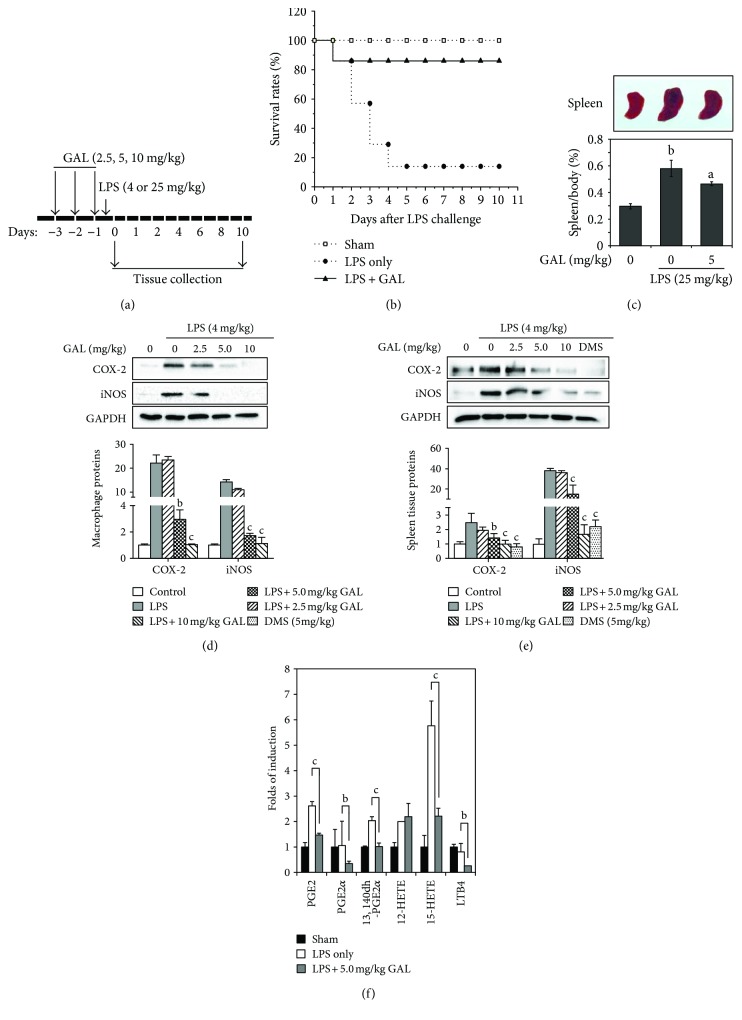
*In vivo* effects of GAL on the survival of mice against LPS-induced toxicity. (a) Experimental design. Mice were treated with GAL, dexamethasone (DMS), or vehicle via i.p. injection for 3 days. On day 3, LPS or 0.9% NaCl saline was administrated via i.p. injection at 12 h after GAL injection. (b) Animal survival. Mice were randomly divided into three groups (*n* = 7): control, receiving vehicle; LPS, receiving 25 mg/kg LPS only; and LPS + GAL, receiving three injections of 5 mg/kg GAL and a single injection of 25 mg/kg LPS. Animal survival was monitored every 12 h for 10 days. (c) Measurement of body weight and spleen size. Following a 10-day treatment, mice were weighed and sacrificed. Spleens were collected, weighed, and imaged. ^a^ *p* < 0.01 (LPS versus control); ^b^ *p* < 0.05 (GAL + LPS versus LPS). (d) Protein expression levels of iNOS and COX-2 in residential peritoneal macrophages. Residential peritoneal macrophages were isolated and analyzed for iNOS and COX-2 levels by Western blotting. Data represent mean ± SD of three animals per group. ^b^ *p* < 0.01 and ^c^ *p* < 0.001 (GAL + LPS versus LPS alone). (e) Protein expression levels of iNOS and COX-2 in spleens. Spleen tissues were recovered and analyzed for iNOS and COX-2 levels by Western blotting. Data represent mean ± SD of three animals per group. ^b^ *p* < 0.01 and ^c^ *p* < 0.001 (GAL + LPS, DMS + LPS versus LPS alone). DMS: dexamethasone. (f) Plasma levels of proinflammatory lipid mediators. Lipid mediators were purified from plasma samples and quantified by LC-MS/MS technique. Data represent mean ± SD of three animals per group. ^b^ *p* < 0.01 and ^c^ *p* < 0.001 (GAL + LPS versus LPS).

To examine the *in vivo* anti-inflammatory efficacy of GAL, we pretreated mice with GAL at three different doses for 3 days prior to LPS stimulation as outlined in Figure 4(a). Firstly, we evaluated the protective effect of GAL on the survival of mice against LPS toxicity. Following the treatments with LPS and GAL, alone or in combination, we monitored the well-being and survival rate of mice over a period of 10 days. As summarized in Figure 4(b), LPS alone killed 85.7% of animals over the period of 5 days, whereas GAL pretreatment at the dose of 5 mg/kg protected 85.7% of animals against LPS toxicity over a period of 10 days. Secondly, we measured the body weight and spleen size of animals at the end of drug treatment. As shown in Figure 4(c), GAL effectively prevented LPS-induced enlargement in spleen size. Thirdly, we compared the expression of COX-2 and iNOS in primary peritoneal macrophages and spleen tissues in response to LPS alone or in combination with GAL. As shown in Figures 4(d) and 4(e), LPS administration (4 mg/kg, i.p.) dramatically increased the protein levels of COX-2 and iNOS in peritoneal macrophages and spleen tissues, whereas GAL pretreatment for three days diminished LPS-induced iNOS and COX-2 expression in a dose-dependent manner. GAL had similar inhibition effect to dexamethasone, a positive drug (shown in Figure 4(e)). Finally, we determined the plasma levels of proinflammatory lipid mediators in mice following the treatments with vehicle, LPS, and GAL + LPS. Lipid mediators were quantified by lipidomics profiling on LC/MS/MS system while standard compounds were used as reference. As shown in Figure 4(f), LPS dramatically increased the production of proinflammatory lipid mediators, for example, PGE2, 13, 14-dh-PGF2*α*, and 12-HETE. GAL pretreatment at the dose of 5 mg/kg effectively antagonized the effects of LPS on the production of proinflammatory lipid mediators including PGE2, PGF2*α*, LTB4, and 12-HETE.

### 3.5. GAL Attenuated LPS-Induced ROS Production and Activation of MAP Kinases

**Figure 5:**
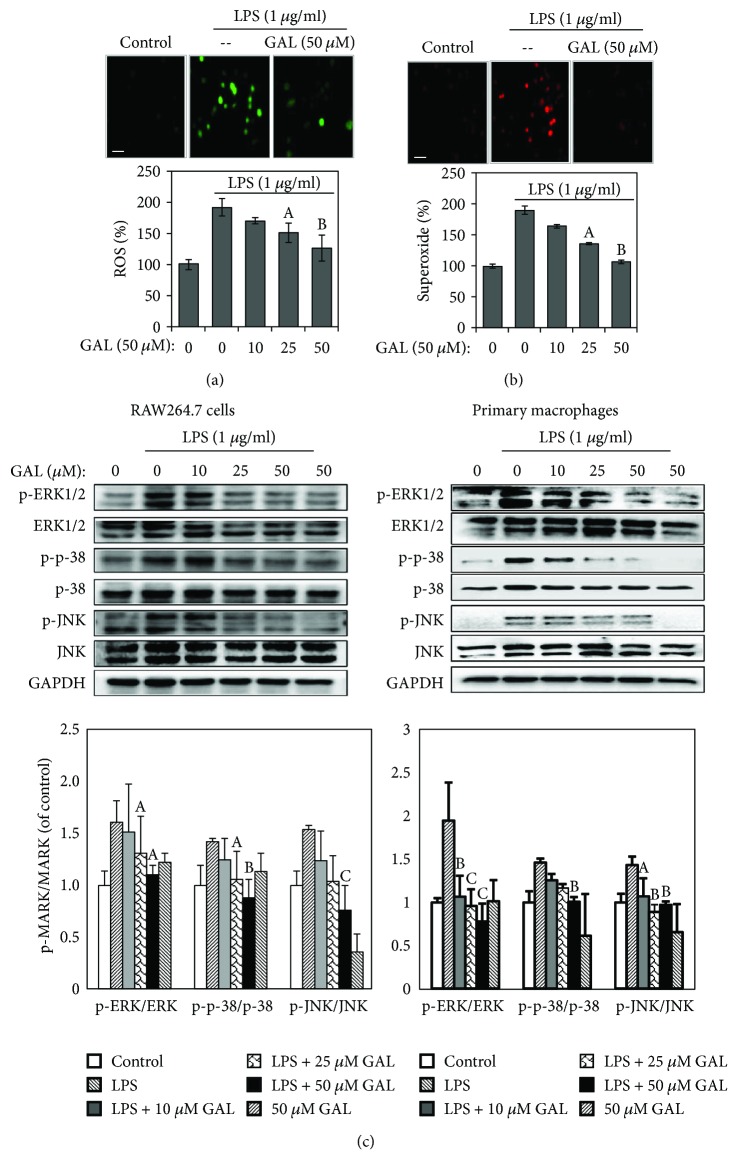
Effect of GAL on LPS-induced ROS production and MAP kinase activation. (a) Detection of intracellular superoxide ion. Following GAL pretreatment for 2 h and LPS stimulation for another 2 h, RAW264.7 cells were incubated with DHE and imaged under a fluorescence microscope (Carl-Zeiss, Jena, Germany). The fluorescence was quantified by using NIH Image J software (http://imagej.nih.gov/ij/). Results represent means ± SD of three independent experiments. ^a^ *p* < 0.05; ^b^ *p* < 0.01 (GAL + LPS versus LPS). Scale bar, 20 *μ*m. (b) Detection of intracellular ROS. Following GAL pretreatment for 2 h and LPS stimulation for another 2 h, RAW264.7 cells were incubated with DCFH2-DA and imaged under a fluorescence microscope (Carl-Zeiss, Jena, Germany). The fluorescence was quantified by using NIH Image J software (http://imagej.nih.gov/ij/). Results represent means ± SD of three independent experiments. ^a^ *p* < 0.05; ^b^ *p* < 0.01 (GAL + LPS versus LPS). (c) Western blot analysis of MAP kinase activation. Following GAL pretreatment for 2 h and LPS stimulation for another 20 min, the cellular proteins were isolated from RAW264.7 cells and primary macrophages and subsequently analyzed by Western blotting using specific antibodies. Western blots were quantified by a densitometric method. ^a^ *P* < 0.05; ^b^ *p* < 0.01 (GAL + LPS versus LPS).

To elucidate the mechanisms underlying the anti-inflammatory activity of GAL, we focused on the proinflammatory signaling pathways previously implicated in the regulation of iNOS, COX-2, and 5-LOX expression. In particular, we firstly examine the effects of GAL on ROS production. We determined the contents of intracellular H_2_O_2_ and superoxide ion by measuring DCFH2-DA-derived and DHE-derived fluorescence. As shown in Figures 5(a) and 5(b), LPS stimulated the generation of H_2_O_2_ and superoxide ion by approximately 1.9-fold in RAW264.7 cells relative to the untreated controls. GAL pretreatment attenuated LPS-induced production of H_2_O_2_ and superoxide ion in a concentration-dependent manner.

On the other hand, we examined the effects of GAL on the phosphorylation of MAP kinases (e.g., ERK1/2, JNK, and p38) by Western blot analysis. As shown in Figure 5(c), GAL suppressed the phosphorylation of ERK1/2 and JNK, p38 MAPK in LPS-stimulated RAW264.7 macrophages in a concentration-dependent manner. GAL also exhibited similar activity in LPS-stimulated primary peritoneal macrophages.

### 3.6. GAL Inhibited LPS-Induced Activation of NF-*κ*B and AP-1

**Figure 6:**
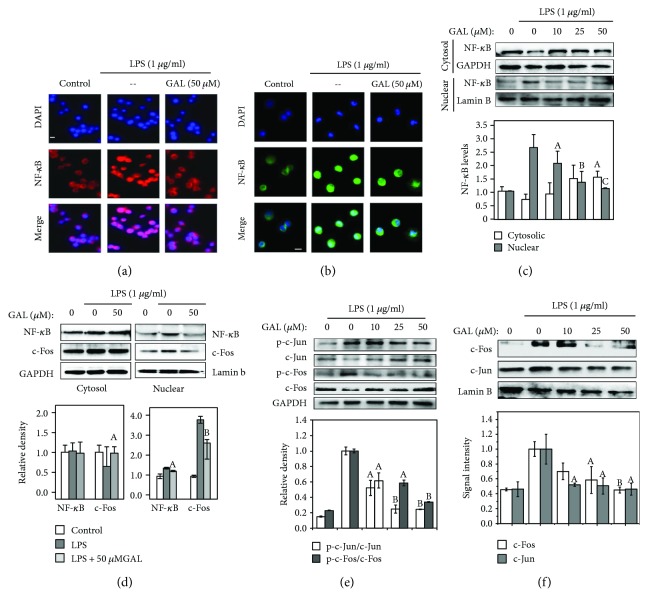
Effects of GAL on LPS-induced activation of NF-*κ*B and AP-1 pathways. (a) Immunofluorescence staining of intracellular NF-*κ*B subunit p65 in RAW264.7 cells. Following the treatments of RAW264.7 cells with GAL for 2 h and LPS for 2 h, NF-*κ*B p65 subunit was stained by specific antibodies, whereas the nuclei were stained by DAPI. Images were captured under a fluorescence microscopy (Carl-Zeiss, Jena, Germany). (b) Immunofluorescence staining of intracellular NF-*κ*B subunit p65 in primary macrophages. Primary peritoneal macrophages were similarly treated with GAL and LPS. NF-*κ*B p65 subunit was detected and imaged as described in (a). (c) Western blot analysis of intracellular NF-*κ*B subunit p65. After the treatments of RAW264.7 cells with GAL and LPS, cytosolic and nuclear proteins were isolated, analyzed by Western blotting, and determined by a densitometric method. The values represent means ± SD of three independent experiments. ^a^ *p* < 0.05; ^b^ *p* < 0.01 (GAL + LPS versus LPS). (d) Western blot analysis nuclear of NF-*κ*B and c-fos in primary macrophages. Primary peritoneal macrophages were similarly treated with GAL and LPS. NF-*κ*B and c-fos were determined as described in (c). (e) Western blot analysis of c-fos and c-jun phosphorylation. After the treatments of RAW264.7 cells with GAL and LPS, the cellular proteins were analyzed by Western blotting with specific antibodies. The protein levels were determined by a densitometric method. The values represent means ± SD of three independent experiments. ^a^ *p* < 0.05; ^b^ *p* < 0.01 (GAL + LPS versus LPS). (f) Western blot analysis of c-fos and c-jun in the cell nucleus. After the treatments of RAW264.7 cells with GAL and LPS, the nuclear proteins were isolated and analyzed by Western blotting with specific antibodies. The protein levels were determined by a densitometric method. The values represent means ± SD of three independent experiments. ^a^ *p* < 0.05; ^b^ *p* < 0.01 (GAL + LPS versus LPS). LB: lamin B.

To further characterize the anti-inflammatory activities of GAL, we focused on the involvement of two well-recognized proinflammatory transcription factors NF-*κ*B and AP-1. Firstly, we examined the effect of GAL on the nuclear translocation of NF-*κ*B p65 subunit by immunostaining. In our experiments, RAW264.7 cells were pretreated with GAL for 2 h and stimulated with LPS for another 2 h. The intracellular NF-*κ*B p65 subunit was stained with specific antibody. We found that GAL effectively prevented the deposition of NF-*κ*B p65 subunit in the cell nuclei in response to LPS stimulation. As shown in Figure 6(a) and Figure 6(b), GAL exhibited similar activity against LPS in RAW264.7 cells and primary macrophages. Secondly, we verified the inhibitory effect of GAL on the nuclear translocation of NF-*κ*B by Western blotting technique. Following the treatments with vehicle, LPS, or LPS + GAL, the cellular proteins were fractionated into cytosolic and nuclear fractions. Based on Western blot analysis in Figure 6(c), GAL suppressed LPS-induced nuclear translocation of NF-*κ*B p65 subunit in a concentration-dependent manner. Thirdly, we investigated the effect of GAL on the phosphorylation and nuclear translocation of heterodimer transcription factor AP-1 involving c-fos and c-jun. Following the drug treatment, we employed Western blotting technique to determine the levels of c-fos, c-jun, phospho-c-fos, and phospho-c-jun. As shown in Figure 6(e), GAL suppressed LPS-induced phosphorylation of c-jun and c-fos in a concentration-dependent manner. The nuclear translocation of c-fos and c-jun was also analyzed by Western blotting.  Figure 6(f) showed that GAL suppressed LPS-induced nuclear translocation of c-jun and c-fos in a concentration-dependent manner. Finally, we compared the effects of GAL on the activation of NF-*κ*B and AP-1 pathways. As shown in Figure 6(d) in primary macrophages, GAL (50 *μ*M) exhibited inhibition of LPS-induced nuclear translocation of NF-*κ*B p65 subunit and c-fos up to approximately 25% and 30%. In addition, we further clarified whether GAL could mimic NF-*κ*B inhibitor and AP-1 inhibitor against LPS signaling. In practical, the cells were treated with LPS alone or in combination with GAL, NF-*κ*B inhibitor, or AP-1 inhibitor. The total RNAs were analyzed by RT-PCR technique for the expression of iNOS, COX-2, and 5-LOX mRNAs. We confirmed that GAL, NF-*κ*B inhibitor, and AP-1 inhibitor showed similar activities against the stimulatory effect of LPS on the induction of iNOS, COX-2, and 5-LOX in macrophages (see Supplementary Fig.  2).

## 4. Discussion

We previously reported the synthesis of GAL as a novel gallic acid derivative and the induction of LTB4DH expression for enhancing macrophage phagocytosis [15]. The present study was designed to evaluate the anti-inflammatory activity of GAL against LPS toxicity in cell culture and mouse model of sepsis. We first established the *in vitro* and *in vivo* effects of GAL on the generation of proinflammatory NO and lipid mediators via suppressing COX-2 and iNOS expression against LPS stimulation. We further elucidated the effects of GAL on ROS production, MAPK signaling pathways, and transcriptional activity of NF-*κ*B and AP-1.

Macrophage plasticity and polarization play critical roles in innate immunity against microbial infection and tissue injury [22]. M1 macrophages are classically activated to induce inflammation and phagocytize pathogenic microbes or apoptotic cells [23]. The weapons of M1 macrophages include proinflammatory cytokines, lipid mediators, and toxic ROS/RNS [24]. To induce macrophage M1 polarization, we employed LPS to challenge macrophages in cell culture system and animal model of endotoxic shock. Our results demonstrated that LPS dramatically increased the production of proinflammatory NO and lipid mediators (e.g., PGE2, TXB2, and LTB4) in macrophages and mouse model. One of the key findings from the present study was that GAL not only diminished the production of proinflammatory enzymes (e.g., COX-2 and iNOS) and the potential mediators (e.g., NO, PGE2, TXB2, LTB4) but also enhanced the survival of animals against LPS toxicity. On the other hand, the activated macrophages produce various chemokines/chemoattractant factors for the recruitment of new polymorphonuclear leukocytes/macrophages [25]. Thus, we examined the effects of GAL on the chemotactic potential of macrophages in Transwell format. We first prepared the conditioned media from macrophage cell culture after exposure to LPS alone or in combination with GAL. We subsequently assayed the cell culture media for the activity to induce the migration of macrophages through Transwell membranes. Our results demonstrated that GAL effectively suppressed LPS-induced chemotaxis of macrophages. Presumably, GAL reduced the production of chemoattractant factors in LPS-stimulated macrophages.

NF-*κ*B and AP-1 are two key transcriptional factors in the regulation of proinflammatory responses in macrophages [26]. It is well-known that both ROS overproduction and MAP kinases (e.g., ERK, JNK and p38) regulate the activation of NF-*κ*B and AP-1 in response to LPS stimulation in macrophages [27–29]. Upon activation, transcription factors NF-*κ*B and AP-1 translocate to the cell nucleus and subsequently induce the expression of proinflammatory enzymes including COX-2, 5-LOX, and iNOS [30, 31]. In the present study, we found that GAL not only attenuated ROS overproduction and the stimulatory effects of LPS on the phosphorylation of MAP kinase (e.g., ERK, JNK, and p38) in LPS-stimulated macrophages but also diminished the activation of NF-*κ*B and AP-1 in LPS-stimulated macrophages. Our results confirmed that GAL effectively suppressed LPS-induced nuclear translocation of NF-*κ*B p65 subunit and AP-1 (i.e., c-fos and c-jun) in a dose-dependent manner. GAL may mimic NF-*κ*B inhibitor and AP-1 inhibitor and thereby downregulate COX-2 and iNOS expression.

Our previous study has reported that GAL also promotes the nuclear translocation of transcriptional factor Nrf2 and the induction of cytoprotective enzyme heme oxygenase-1 (HO-1) [15]. It is interesting to note that the activation of Nrf2/HO-1 pathway may inhibit NF-*κ*B and AP-1 and thereby diminish the production of proinflammatory mediators [32]. However, timing appears to be critical for GAL to exhibit its activities against either Nrf2/HO-1 pathway or NF-*κ*B and AP-1 pathways. In our experiments, GAL inhibited LPS-induced activation of NF-*κ*B and AP-1 pathways over a period of 3 h, whereas GAL induced Nrf2 translocation and HO-1 expression after 6 h treatment. Therefore, GAL may suppress LPS-induced activation of NF-*κ*B and AP-1 pathways mainly via inhibiting ROS formation and MAP kinases.

**Figure 7:**
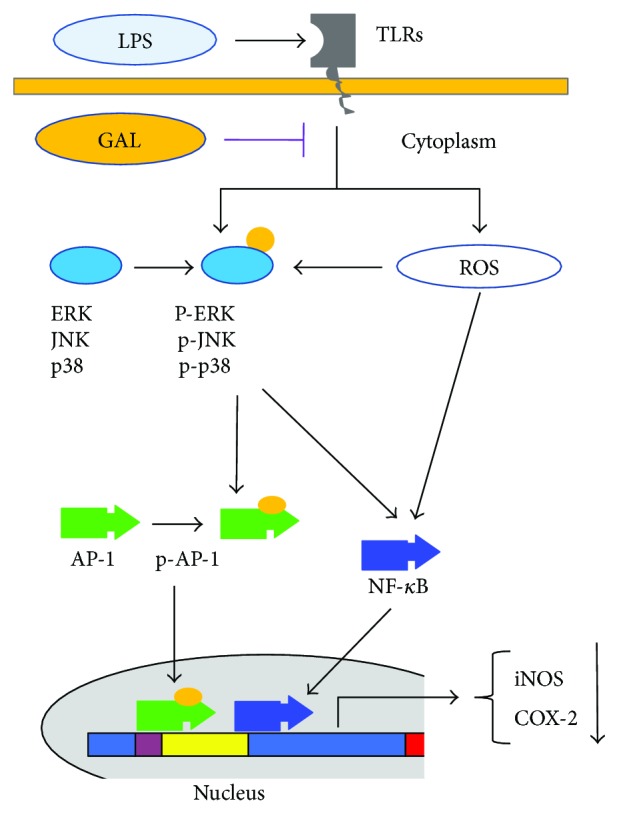
Proposed mechanisms underlying the anti-inflammatory effects of GAL. GAL may primarily target the production of ROS and the activation of MAP kinases in the upstream of proinflammatory signaling pathways.

In summary, we investigated the anti-inflammatory activities of the new in-house product GAL conjugate in the present study. Our results suggest that GAL may exhibit anti-inflammatory activities via the mechanisms as outlined in Figure 7. GAL initially interrupts LPS-induced production of ROS and activation of MAP kinases (i.e., ERK, p38, and JNK). Subsequently, GAL antagonizes the effects of LPS on the nuclear translocation of transcription factors NF-*κ*B and AP-1, causing significant reduction of COX-2 and iNOS expression. As result, GAL may protect mice against LPS toxicity via controlling the generation of proinflammatory lipid mediators and toxic NO. Thus, we anticipate that GAL will serve as a useful lead for the development of novel anti-inflammatory drugs against endotoxic shock.

## Abbreviations

AP-1: Activating protein-1
BOP: Benzo-triazol-1-yloxy-tris(dimethylamino)phosphonium hexafluorophosphate
ECL: Enhanced chemiluminescence
COX-2: Cyclooxygenase-2
CYP450: Cytochrome P450
DAPI: 4′,6-diamidino-2-phenylindole
DCFH2-DA: 2′,7′-dichlorofluorescein-diacetate
DHE: Dihydroethidium
DMEM: Dulbecco's modified Eagle's medium
DMSO: Dimethyl sulfoxide
ESI-MS: Electrospray ionization-mass spectrometry
GAL: Gallic acid-L-leucine conjugate
GAPDH: Glyceraldehydes-3-phosphate dehydrogenase
GRF: General Research Fund
HEPES: N-2-hydroxyethylpiperazine-N′-2-ethanesulfonic acid
HRP: Horseradish peroxidase
IgG: Immunoglobulin G
iNOS: Inducible nitric oxide synthase
JNK: c-Jun N-terminal kinases
LPS: Lipopolysaccharide
LOX: Lipoxygenase
LTB4: Leukotriene B4
MTT: Benzo-triazol-1-yloxy-tris(dimethylamino)phosphonium hexafluorophosphate
NF-*κ*B: Nuclear factor-kappa B
NO: Nitric oxide
PGE1: Prostaglandin E1
PGE2: Prostaglandin E2
PGE3: Prostaglandin E3
PGF2*α*: Prostaglandin F2*α*
TXB2: Thromboxane B2
PLA2: Phospholipase A2.

## Supplementary Materials

Supplementary Materials Supplementary Figure 1 shows effects of gallic acid (GA), methyl-l-leucine (L-Leu), and GAL on LPS-induced COX-2, 5-LOX, and iNOS in RAW264.7 cells. Supplementary Figure 2 indicates parallel analysis of GAL, NF-*κ*B inhibitor, and AP-1 inhibitor for the suppression of COX-2, iNOS, and 5-LOX mRNA expression. Supplementary Figure 3 shows the MS profiles for lipid mediator detection. Supplementary Table 1 demonstrates the parameters of LC/MS/MS under MRM for lipid mediators.
