# Supplementary material for: Gallic Acid-L-Leucine Conjugate Protects Mice against LPS-Induced Inflammation and Sepsis via Correcting Proinflammatory Lipid Mediator Profiles and Oxidative Stress
Source: Oxid Med Cell Longev. 2018 Mar 25;2018:1081287. doi: 10.1155/2018/1081287 (PMC5889890; doi:10.1155/2018/1081287)
Supplement: Supplementary Materials — Supplementary Figure 1 shows effects of gallic acid (GA), methyl-l-leucine (L-Leu), and GAL on LPS-induced COX-2, 5-LOX, and iNOS in RAW264.7 cells. Supplementary Figure 2 indicates parallel analysis of GAL, NF-κB inhibitor, and AP-1 inhibitor for the suppression of COX-2, iNOS, and 5-LOX mRNA expression. Supplementary Figure 3 shows the MS profiles for lipid mediator detection. Supplementary Table 1 demonstrates the parameters of LC/MS/MS under MRM for lipid mediators. [file 1081287.f1.pdf]

Supplementary File

Gallic acid-L-leucine conjugate protects mice against LPS-induced inflammation and sepsis via correcting pro-inflammatory lipid mediator profiles and Oxidative stress

Yuanyuan Cheng[1], Xuechen Li[2], Hung-Fat Tse[3], Jianhui Rong[1,*]

[1]School of Chinese Medicine, The University of Hong Kong, Hong Kong, China

[2]Department of Chemistry, The University of Hong Kong, Hong Kong, China

[3]Department of Medicine, The University of Hong Kong, Hong Kong, China

*Corresponding author: jrong@hkucc.hku.hk

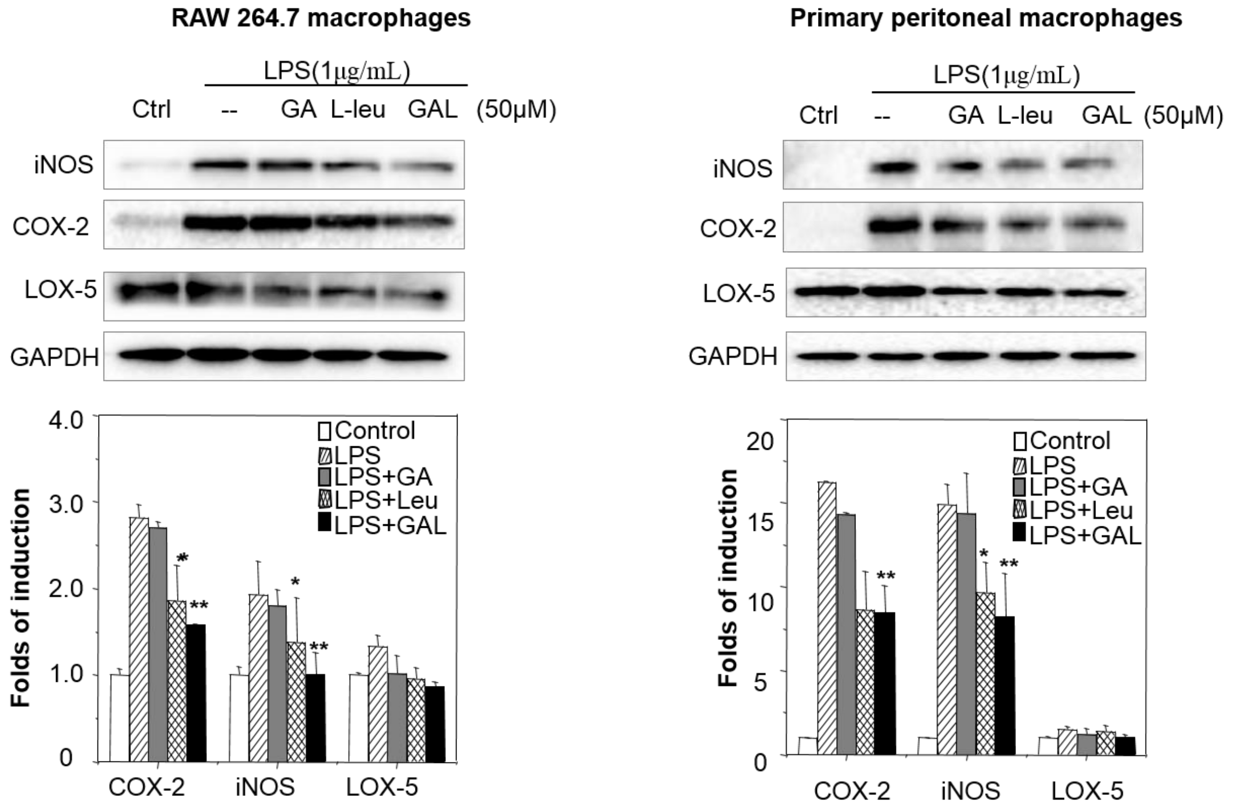

Supplementary Figure 1. (A) Effects of Gallic acid (GA), methyl-l-leucine (L-Leu) and GAL on LPS-induced COX-2, 5-LOX and iNOS in RAW264.7 cells. Following 24 h treatment with GAL and LPS, the expression of COX-2, 5-LOX and iNOS was analyzed by Western blotting, and quantified by a densitometric method. The results were expressed as a percentage of the untreated control (n=3). *, $p<0.05$; **, $p<0.01$ (Sample vs LPS alone). (B) Effects of Gallic acid (GA), methyl-l-leucine (L-Leu) and GAL on LPS-induced COX-2, 5-LOX and iNOS in primary macrophages. The expression of COX-2, 5-LOX and iNOS was analyzed in the same fashion as described in Panel B. The results were expressed as a percentage of the untreated control (n=3). **, $p<0.01$; ***, $p<0.001$ (Sample vs LPS alone).

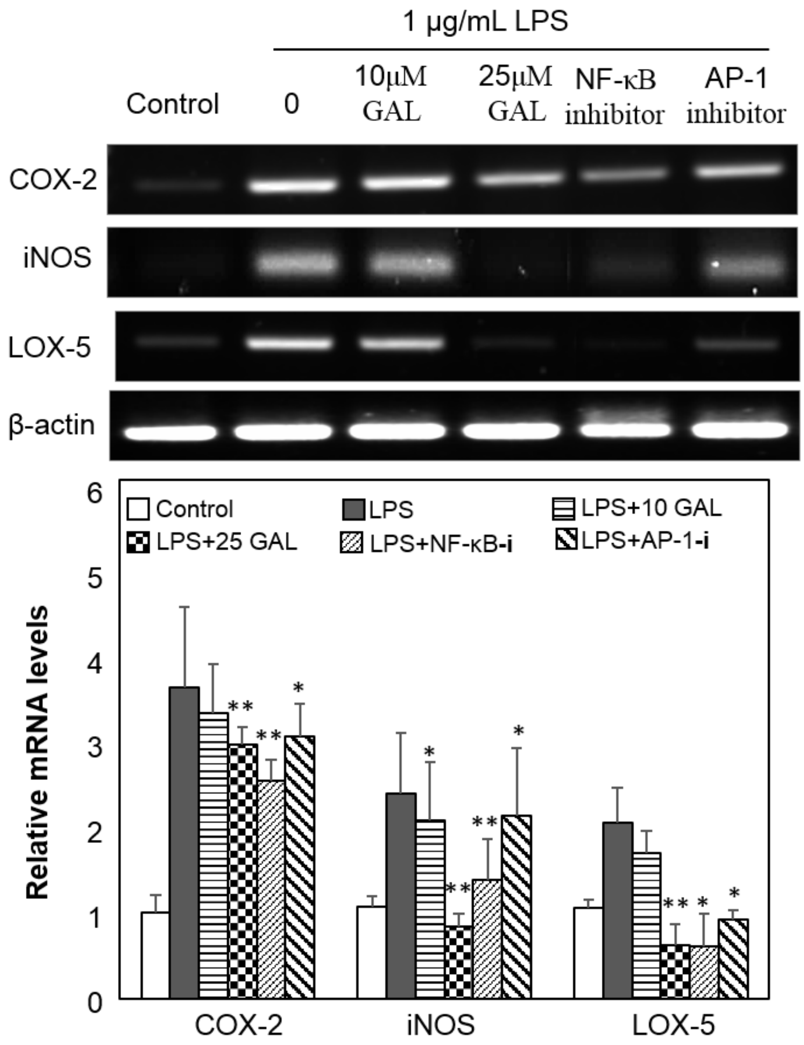

Supplementary Figure 2. Parallel analysis of GAL, NF-kB inhibitor and AP-1 inhibitor for the suppression of COX-2, iNOS and 5-LOX mRNA expression. (A) RT-PCR detection of COX-2, 5-LOX and iNOS mRNA expression. Following 24 h treatment with GAL (10 and 25 μM), NF-κB inhibitor (10 μg/mL), AP-1 inhibitor (1 μM) and LPS stimulation, the total RNAs were isolated from RAW264.7 cells, and analyzed for COX-2, 5-LOX and iNOS mRNA expression by RT-PCR. The gels were quantified by a densitometric method. The results were expressed as a percentage of the untreated control (n=3). *, $p<0.05$; **, $p<0.01$ (GAL+LPS vs LPS).

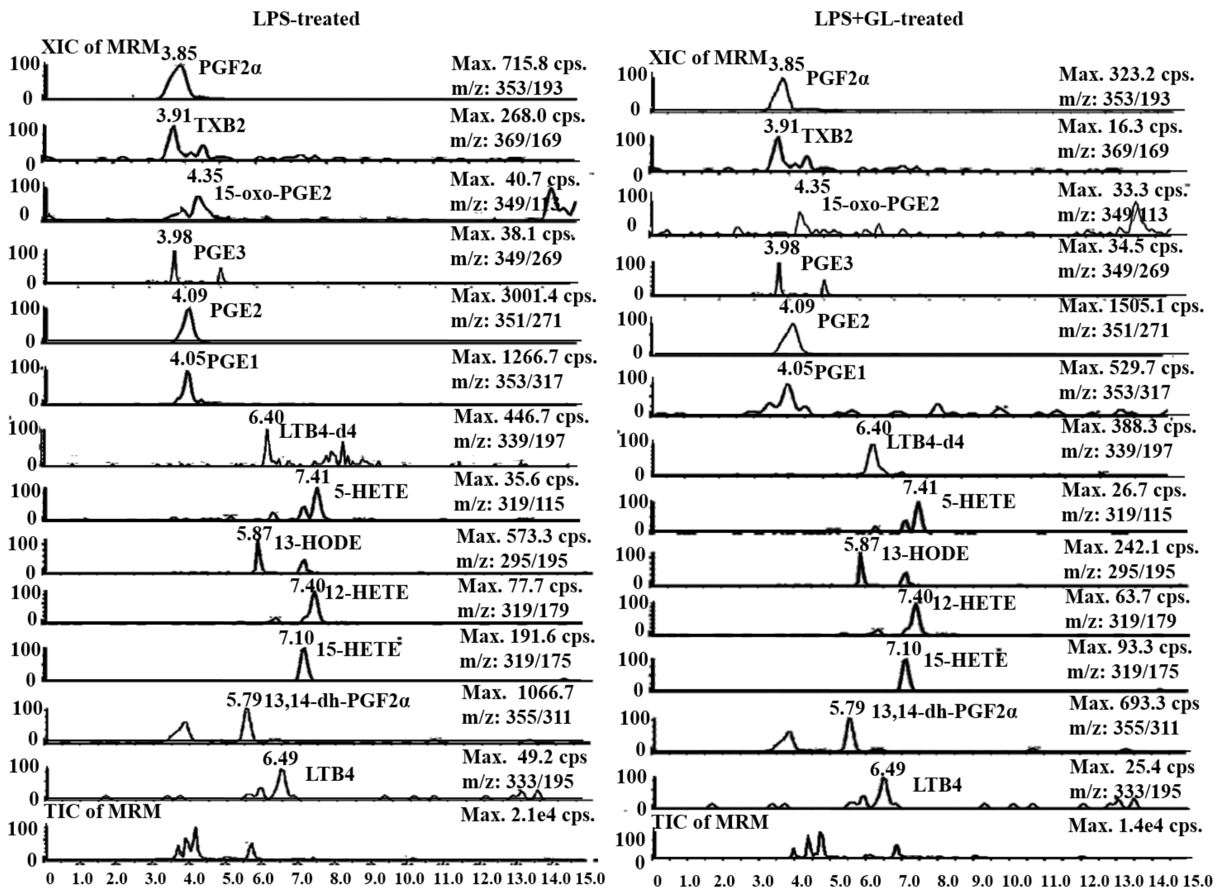

Supplementary Figure 3. The MS profiles for lipids mediator detection.

Table 1. The parameters of LC/MS/MS under MRM for lipids mediators.

**Lipid mediator**	**MRM**	**CE(eV)**	**RT (time)**
PGE2	351 -- 271	17	4.09
PGE1	351-- 271	17	4.05
PGF2α	353 -- 193	25	3.85
PGE3	349 -- 269	15	3.98
15-oxo-PGE2	349 -- 113	20	4.35

13,14-dh-PGE2α	355 -- 311	25	5.79
TXB2	369 -- 169	25	3.91
5-HETE	319 -- 115	20	7.41
12-HETE	319 -- 179	17	7.40
15-HETE	319 -- 175	18	7.10
13-HODE	295 --195	25	5.87
LTB4	335 -- 195	17	6.48
LTB4-d4	339 -- 197	17	6.47
